# Advances in electroactive materials-based strategies for promoting skin wound healing

**DOI:** 10.3389/fmedt.2026.1919503

**Published:** 2026-09-28

**Authors:** Haihong Ge, Xi Ren, Zeyuan Lei

**Affiliations:** Department of Plastic and Cosmetic Surgery, Xinqiao Hospita, Army Medical University, Chongqing, China

**Keywords:** conductive hydrogels, electrical stimulation, electroactive materials, endogenous electric fields, self-powered materials, smart dressings, wound healing

## Abstract

The loss of homeostasis of the local microenvironment is one of the processes encountered in chronic wound repair and is therefore a big challenge in clinical practice. Endogenous electric fields, being important biophysical cues in the wound bed, guide the direction of movement of the reparative cells, control the inflammatory process and help in the regeneration of the tissues. The classical external electrical stimulation equipment is strongly limited in the portability that can be offered and in the biocompatibility that can be ensured, which limits the wider clinical application of electroceutical approaches for wound care. Some recent developments in the field of electroactive materials give new methodologies for wound therapy by using electrical stimulation. In this article, first, the physiological process of wound closure and the origin and the properties of the endogenous electric fields are summarized. Then, it concentrates on three types of electroactive materials: conductive hydrogels that allow the effective conduction and enhancement of electrical signals through flexible interfaces; self-powered materials that generate their own power, e.g., by means of piezoelectricity or triboelectricity, transforming ambient mechanical energy into therapeutic electrical fields; and multifunctional composite materials that combine electrical stimulation together with antibacterial, exudate control, regulated drug delivery and immunomodulatory functions in a single system. Then the review explains in an ordered fashion the different mechanisms how the electric field can help wound repair, from the generation of directional signaling cues to the modulation of the inflammatory, proliferative and remodeling stages and also to the antimicrobial actions. Finally, it discusses existing barriers, i.e., the conflict between standard and personalized electrical stimulation parameters, the lack of long-term durability assessments and comprehensive biosafety evaluations. The review ends with some outlooks on the progress of future integrated closed-loop intelligent repair systems with sensing, decision-making and responsive functionalities.

## 1 Introduction

If skin injuries are not handled in a timely and appropriate way, they could become infected, develop into chronic ulcers, or even tissue necrosis. This increases the suffering and medical burden on patients (1, 2). The aging population and the increase in chronic conditions, such as diabetes, result in a constant rise in the incidence of hard-to-heal wounds (3, 4). In the US, more than six million people suffer from non-healing wounds, and the prevalence is estimated at around 3% in the elderly, with annual medical costs exceeding 50 billion US dollars (5, 6). Such chronic wounds do not heal within four to six weeks because the healing is often stuck in the inflammatory stage (7). Several factors affect wound healing, including the cause, location, size and depth of the wound, as well as the patient's general condition, such as diabetes and circulatory problems (8). A typical example is the diabetic foot ulcer, which is often accompanied by persistent infection, chronic inflammation, local hypoxia, and poor angiogenesis, which together delay wound healing (9). In these non-healing wounds, the immune environment is characterized by a constant presence of pro-inflammatory M1 macrophages and no shift to the reparative M2 state (10). The existing treatment approaches mainly include the use of topical drugs and biological agents, the use of vacuum-assisted closure, the transplantation of engineered skin substitutes, the use of hyperbaric oxygen, and the use of therapies with growth factors or cells (11). Although these methods are very promising, they are often associated with high costs, variable clinical outcomes, and unresolved questions regarding their biological safety. Therefore, there is an urgent need to develop innovative treatment approaches that are not only more effective, but also more economical.

Electrical stimulation treatment helps wound recovery because it generates electrical fields at the injury site, and it has received growing scientific interest in the last twenty years (12). A large amount of work shows that electrical fields with given characteristics can accelerate the directed movement of cells from the periphery of the wound to its center, increase the multiplication and specialization of the cells, and stimulate the formation of new blood vessels. As a consequence, the healing time is lower, while an excessive inflammatory response and scar contraction are reduced (13–15). A recent meta-analysis of 10 randomized controlled trials (451 patients with diabetic ulcers) demonstrated that electrical stimulation, with electrodes placed directly on or around the wound, significantly improved ulcer healing rates compared with standard care alone (RR 1.45, 95% CI: 1.18–1.78, *p* < 0.001) (16). However, the traditional electrical stimulation treatment on the other hand, needs an external power, needs complicated procedures and is not easy to be portable, the three factors are in total the less of the freedom of the patient movement. In recent years, with the progress of materials science and bioengineering, new classes of electroactive materials—such as conductive hydrogels, self-powered materials, and integrated multifunctional electroactive materials—have been developed. These advances can make the therapeutic device be more portable and also improve the wound healing environment with the cooperation effects between the materials and the electrical fields, they give the new possibilities in this area.A critical consideration in the design and application of these materials is the relationship between electrical stimulation parameters—including field strength, waveform, frequency, duration, and electrode configuration—and the specific biological responses they elicit in different cell types. The efficacy of electrical stimulation is highly parameter-dependent, and the heterogeneity of reported stimulation protocols across studies has hindered the establishment of standardized guidelines for clinical translation (17). Understanding how these parameters translate into cellular behaviors is therefore essential for rational material design and for bridging the gap between *in vitro* observations and *in vivo* wound healing applications. In this article, the design ideas and the research developments related to the above material approach are reviewed methodically, we intend to give a footing of following studies.

## 2 Skin wound healing and the basis of electric fields

### 2.1 Physiological process of wound healing

The process by which the skin injuries repair themselves is a series of dynamic, interlaced and overlapping stages, which are usually divided in hemostasis, inflammation, proliferation and tissue remodeling (18, 19). When the tissue is damaged, the coagulation system of the body is activated and a fibrin-based clot is created that will incorporate the aggregated platelets; such a structure will serve to stop the ongoing hemorrhage and to create a temporary scaffold that will allow the movement of the cells involved in repair. A few days after the injury the inflammatory stage starts, during which cells like neutrophils, macrophages and lymphocytes go to the wound site in a sequential way that will allow getting rid of infective agents and dead tissue. Tumor necrosis factor-α (TNF-α), interleukin-1 (IL-1) and transforming growth factor-*β* (TGF-*β*) are the main signaling molecules that are important for the regulation of those activities. After that the proliferative phase starts, involving the multiplication and movement of fibroblasts and the process of re-epithelialization that is carried out by keratinocytes; at the same time, the endothelial cells start to form the first capillary networks to deliver oxygen and other essential nutrients to the nascent granulation tissue (18, 19). After a period of roughly two to three weeks, the healing process goes into the remodeling stage. In this stage the freshly laid down collagen fibers go through a slow process of cross-linking and realignment. At the same time the type III collagen is slowly replaced by the type I collagen. This series of changes work to reestablish the structural strength and integrity of the skin (18, 19).

If the normal progression of these biological events is disturbed, then a chronic wound is formed. Such wounds do not heal in the normal four- to six-week period following injury in general, as the healing mechanism is stopped during the inflammatory stage (7). One of the hallmarks of chronic wounds is the disturbance of the local wound environment. This is characterized by an excess of wound fluid, presence of bacteria still ongoing, shortage of growth factors that are required, and continuous presence of M1 macrophage that do not properly switch into the M2 state (10, 20, 21). Underlying systemic problems, like diabetes, can cause for example peripheral vascular disorders and nerve damage. These decrease the blood flow and the feeling in the wounded area, which worsens the local tissue oxygen deprivation, lack of blood supply and is more susceptible to infection (22). Therefore, better healing in chronic wounds should be promoted with a strategy that has to be global. It has to combine the management of infection and inflammation, the modulation of local wound conditions, and the encouragement of tissue regeneration.

### 2.2 Discovery and characteristics of endogenous electric fields in wounds

Besides being a shield, the skin also functions as an internal biological source of power. The first information about faint bioelectric currents at the site of a human skin injury was first recorded in the 19th century by the German physiologist Du Bois-Reymond (23). In the 1980s, Barker and co-workers measured a voltage difference of 30 to 100 millivolts on the hairless skin of guinea pigs and proposed the “skin battery” hypothesis (24). Foulds and Barker later found similar voltage measurements for the human skin and remarked that these electrical potentials have consistent variations among different anatomical locations (25).

It is now clear that the intrinsic electrical potential observed on intact skin is essentially a transepithelial potential (TEP). The TEP is built up by the polarized distribution of ion channels and pumps in the apical and basolateral membranes of epithelial cells, together with active ion transport by Na⁺/K⁺-ATPase. This activity generates a negative potential at the skin surface with respect to the tissue below, typically ranging between 10 and 60 mV (26–28). If the skin is cut, the continuity of the epithelial barrier is broken, and there is a local short-circuit of the TEP at the wound site. Ions then flow from the adjacent healthy tissue, through the interstitial pathways, towards the center of the wound. This ionic flow creates a lateral direct-current electric field directed from the wound periphery to its center. In this configuration, the wound edge is the anode and the center the cathode, and the field strength is about 40 to 200 mV/mm (28, 29). *In vivo* electric field imaging by Nuccitelli and co-workers directly confirmed the presence of this electric field, and they measured a strength of 177 ± 14 mV/mm at the edge of a mouse skin wound. This electric field remains at peak intensity for about three days after injury and then decreases in correspondence with the process of re-epithelialization until the wound is healed (30). It is worth noting that the polarity of the transepithelial potential is different in different epithelial tissues: for example, the surface of the corneal epithelium is positively charged with respect to its basal layer. Thus, the lateral electric field produced at corneal injuries is in the opposite direction with respect to that observed at skin wounds. This difference of polarity explains the differences in the direction of movement of the epithelial cells from different sources in electric fields, as we will discuss later (31).

Sodium and chloride ions are the main charge carriers in the wound currents. In a study done on a rat corneal wound model, Reid and others showed that sodium ions are the main ions driving the ion transport in the intact cornea and the leakage current at the centre of the wound. On the other hand, chloride ions contribute more to the endogenous current found at the periphery of the wound. They also showed that if there is a pharmacological intervention that changes the flow of chloride ions, it can increase or decrease the *in vivo* wound healing rate accordingly (32).

The lateral electric field described above can be used to make various types of repair-related cells move. Cellular electrotactic orientation is different for different types of cells: neutrophils and keratinocytes migrate to the cathode, fibroblasts and macrophages move to the anode. For endothelial cells, the direction of electrotaxis is different according to the vascular origin of the cells: the microvascular endothelial cells migrate to the cathode, and the macrovascular endothelial cells migrate to the anode (33). Through gene knockout experiments, Zhao et al. found that phosphatidylinositol-3-OH kinase-*γ* (PI3K*γ*) and phosphatase and tensin homolog (PTEN) are two important regulatory genes in the electrotactic mechanism, and they showed that knocking out PI3K*γ* eliminates the electrotactic behavior, while knocking out PTEN amplifies the electrotactic response (34).

In the condition of chronic wounds, the endogenous electric field is often compromised. With three independent animal models of diabetes, Shen and colleagues showed that the electric field signal of the diabetic corneal injury is significantly weaker than that of the healthy corneal tissue (35). Furthermore, the fibroblasts and macrophages which are isolated from the chronic wound have already functionally changed (36). Whether their reaction to the electric stimulation is the same as that of cells obtained from healthy tissue has not been systematically investigated.

Since the function of the endogenous electric field is so important for the healing of wounds, the externally administered electrical stimulation which mimics or enhances these natural fields is considered a very promising way to promote wound healing. In particular, this is of interest for the case of chronic wounds, where the signals of the body's electric field are smaller (37). The action of such kind of electrical therapy depends on many factors, e.g., the level of the voltage, the setting of the frequency, the pattern of the waveform and so on. Nevertheless, a set of the best parameters is not yet defined for all (17). All these challenges have pushed the scientists to develop new electroactive materials which will produce or transmit the electric fields. Now the wound dressing itself is to play the role of the source or the regulator of the electric fields. The endogenous wound electric field also provides a physiological benchmark for exogenously applied stimulation. The field strengths measured *in vivo*—typically 40–200 mV/mm—overlap substantially with the parameter ranges that have been shown to guide migration of keratinocytes, fibroblasts, and neutrophils *in vitro* (29, 33). This observation suggests that multiple cell types involved in wound repair are inherently responsive to field strengths within this physiological range, a principle that has guided the selection of stimulation parameters in numerous subsequent studies.

## 3 Collaborative strategies of electric fields with novel materials and representative studies

The electroactive materials that we discuss in this review operate through different mechanisms, but they are all designed to influence the wound healing process by means of electrical signals. Based on how they generate or transmit these signals, we group them into three types. The first comprises conductive materials that serve as passive conduits to transmit endogenous electric fields or externally applied signals through flexible, tissue-conforming interfaces. The second group consists of self-powered materials that actively generate therapeutic electric fields from ambient mechanical, thermal, or light energy. The third group includes integrated multifunctional systems that combine electrical functionality with other therapeutic modalities, such as antimicrobial activity, exudate management, controlled drug delivery, or immunomodulation, in a single platform. It should be noted that these categories are not mutually exclusive—many conductive hydrogels also incorporate antimicrobial or drug-delivery functions. The classification here is based on the primary mode of electrical signal generation or transmission.

### 3.1 Conductive hydrogels: An ideal platform for transmitting and amplifying electrical signals

Hydrogels are considered the ideal matrix for developing wound dressings, as they are able to retain a huge amount of moisture, have excellent biocompatibility and mechanical characteristics similar to soft biological tissues. By incorporating electroactive functional elements into the three-dimensional network of the hydrogel, such as conductive polymers (for example polyaniline, polypyrrole and PEDOT:PSS), carbon-derived nanomaterials (for example graphene, carbon nanotubes and MXene), or metal nanoparticles, conductive hydrogels can be prepared, which have the advantages of the hydrogel itself and are also electrically active (38). The choice of the conductive polymer can influence both the electrical conductivity and the processability of the final hydrogel material. Polypyrrole and polyaniline give the best conductivity but in general they are comparatively stiff; on the other hand PEDOT:PSS has gained great attention in recent research because of its good aqueous dispersibility and solution-based processability (39). Among carbon-based nanomaterials, MXene, a class of novel two-dimensional transition metal carbides and nitrides, has rapidly become a focus in the research of hydrogel-based wound dressings, as they have the combination of metallic electrical conductivity and high hydrophilicity (40). However, it is worth remembering one important aspect: MXenes, at least Ti₃C₂Tₓ as a typical example, are easily oxidised in aqueous, oxygen-rich biological environments. In such environments, the surface layer of the transition metal would progressively change to metal oxides, such as TiO₂, within days, and the conductive property would significantly deteriorate. This is a fundamental flaw for ensuring the long-term stability of MXene-containing hydrogels in the wet condition of the healing wound. To prevent this oxidative degradation, different approaches are under investigation. These include the application of surface passivation coatings, adding antioxidant agents, storing in organic solvents, designing the hydrogel matrix to provide protection, and refining the synthesis parameters (41). However, such antioxidant strategies have been validated predominantly in aqueous dispersion *in vitro*, and the retention of MXene conductivity under the enzymatic and oxidative conditions of a full-thickness wound has not been systematically demonstrated over the complete healing period *in vivo*.

These substances are able to efficiently deliver the endogenous electric fields generated within the wound and are also suitable as pliable electrodes to deliver external electrical stimulation. In contrast to the use of rigid metal electrodes, hydrogel-based electrodes are able to make close, shape-conforming contact with the uneven and moist wound surface, and therefore can lower the impedance at the interface between the electrode and the tissue. This characteristic also enables safe electrical stimulation to be delivered at comparatively lower voltages and helps to avoid elevated local current densities which might be the result of not so good contact, and therefore reduce the possible harm to the developing tissue (38). The electrical conductivity of conductive hydrogels should be kept in an appropriate range: if it is too high, charge could be locally built up and unwanted electrochemical byproducts could be formed, and if it is not good enough, the efficient transfer of electrical impulses cannot be achieved. So far, most current research has engineered the hydrogel conductivity in the range between 10⁻^3^ and 10⁻^1^ S/cm. This range is generally considered to approximate the electrical characteristics of viable, hydrated skin tissue (such as the dermis) (42). It should be noted, however, that this benchmark is derived from the dielectric properties of intact, normal skin rather than from direct impedance measurements of wound-bed tissue. Systematic characterization of wound-bed conductivity across different healing stages in human subjects has not yet been reported. Therefore, such hydrogels can act as pliable electrodes able to transmit externally applied electrical stimuli to the wound site (43). For situations in which only the passive transmission of the wound's own endogenous electric field is needed to maintain the electrotactic processes, the specifications of the required conductivity can be less strict. However, the exact correlations of the defined conductivity benchmarks with the different modes of electrical stimulation need further systematic research to be elucidated.

Several recent investigations have explored the potential of conductive hydrogels to promote wound repair through different mechanisms. Concerning the interplay between materials, cells and electrical stimulation, one study has built a 3D conductive hydrogel scaffold seeded with dermal fibroblasts and subjected it to external electrical stimulation. In a rat model with full-thickness skin wounds, the group treated by the combination of the two showed the fastest rate of wound contraction. Moreover, the density of newly generated blood vessels and the level of collagen accumulation were considerably higher than those from any of the single-component treatments (44). Concerning designs that combine antibacterial properties and conductivity, Ren et al. mixed MXene nanomaterials with broad-spectrum antimicrobial peptides to build a nanocomposite hydrogel dressing named GelMA@Ti₃C₂/V-Os. With the application of the dressing together with suitable electrical stimulation, this dressing sped up the healing process, and the final percentage of wound closure was also greater in comparison with both the untreated control and the group treated with a hydrogel that does not contain MXene (45). Yang et al. developed a multifunctional hydrogel dressing named PMQG by crosslinking of acrylic acid, quaternized chitosan and MXene nanosheets. This dressing showed, when tested on a diabetic mouse skin wound model, outstanding capabilities in promoting angiogenesis and speeding up the healing, without the need for complex external stimulation devices (46). Lee et al. fabricated a composite conductive hydrogel from GelMA and PEDOT:PSS by three-dimensional printing. The electrical conductivity and surface microtopography of the material gave bidirectional regulatory effects, which considerably improved the directional migration efficiency of fibroblasts. This provides a new way for the enhanced physicochemical design of materials for wound repair (47).

As shown by the research, by using well-designed material engineering, the conductive hydrogel systems are able to integrate several functions at the location of the injury. These are the conduction of bioelectrical signals, antimicrobial effects, structural reinforcement, and the keeping of a humid microenvironment for healing. Their path toward clinical application however faces several open problems. In the moist conditions of a wound, the hydrogel matrix will swell or slowly degrade, and this will cause a steady decrease of both its electrical conduction capabilities and its structure. Some researchers have tried to improve the mechanical properties of these hydrogels by using dual-network architectures (48). But the problem of how to improve the material stability without losing satisfactory conductivity is still to be well explored. Finally, the toxicological evaluation is still not complete, in particular for the long-term degradation patterns of the conductive element in a living organism and the biological safety of the breakdown byproducts.

### 3.2 Self-powered materials: electrical stimulation strategies based on energy conversion

The problem of transmitting electrical signals has been well solved by the conductive hydrogels; however, the application of external electrical stimulation still relies on a power source and special driving apparatus. The self-powered wound dressings mainly use the piezoelectric or triboelectric effects to directly convert the ordinary human motions, such as the bending of the joint, the stretching of the muscle, and the expansion and contraction of the chest during breathing, and even the very small mechanical vibrations from the environment, into therapeutic electrical fields. It completely does away with the need for an outside power supply during the whole treatment process (49). Taking the basic energy conversion principles into consideration, the self-powered materials used in wound care have developed from early piezoelectric and triboelectric nanogenerators to a wider variety of types, such as thermoelectric, photovoltaic, capacitive and bioelectric materials (50).

In the field of piezoelectric materials, a considerable amount of research attention has been given to organic piezoelectric polymers, such as polyvinylidene fluoride (PVDF) and its copolymers, due to their favorable mechanical flexibility and processability (51). For example, a research group headed by Yang et al. has fabricated a pliable, self-powered piezoelectric film with a poly(vinylidene fluoride-trifluoroethylene) copolymer, P(VDF-TrFE), with the additive of tetrabutylammonium chloride (TBAC). Adding TBAC improves the ferroelectric and piezoelectric performance of the composite and the output voltage is increased roughly 5.3 times relative to the P(VDF-TrFE) polymer without the modifier. In application, when this film was placed at the wound location, it could automatically generate an electric field in response to the minor mechanical strains caused by body motion. In the rat model of skin wound healing, this method can make the wound almost fully closed by the seventh day (52).

In the domain of triboelectric nanogenerators, Zhang and coworkers fabricated a wearable patch based on an ionic triboelectric nanogenerator (iTENG). This system is able to convert the mechanical energy of human body movement to electrical power. It also uses the internally generated electric field to provide quantitative and demand-coupled delivery of particular therapeutic metal ions, such as Zn^2+^, in the patch. When it is put in a diabetic mouse model with skin wounds, the iTENG patch has great antibacterial properties, stimulates new blood vessel formation, increases collagen accumulation and speeds up wound healing (53). As a class, triboelectric nanogenerators are known to produce open-circuit voltages in the range of tens to hundreds of volts and short-circuit currents in the microampere range (49). For example, a dual-sided TENG-based hydrogel patch generated 57 ± 5 V and 2.2 ± 0.3 *μ*A (54). A field strength of 40–200 mV/mm across a wound distance of 1–5 mm corresponds to only 0.04–1 V. TENG outputs are therefore 50 to 1000 times higher than the voltages that optimally guide cell migration, explaining why power management circuits are essential for clinical translation.

Besides that, the technology of implantable photovoltaic energy harvesting, which converts light to electricity, is also one of the important methods for energy conversion. It has shown great promise for promoting light activated, self powered wound healing materials (55). For example, Han and co-workers demonstrated a microelectromechanical system (MEMS) electrode array with wearable organic solar cells. When illuminated by sunlight and near infrared light, it generates an electrical field. Both the laboratory tests and the studies with the diabetic mouse model confirmed that the system promoted the growth and the multiplication of skin fibroblasts, and also increased the production of transforming growth factor (TGF) (56). However, the reliance on external light sources introduces practical constraints, such as light attenuation by overlying wound dressings and variability in wound bed illumination, which remain to be addressed before clinical deployment.

One of the main problems that self-powered materials meet is the mismatch between the sporadic character of the energy collection and the biological tissue which needs a constant stimulation. The mechanical energy coming from human motion is random, of low frequency and with a large variation of the intensity, so that the piezoelectric or triboelectric outputs are pulsating. Moreover, the raw outputs are typically high-voltage transient signals that differ substantially from the low-voltage direct-current endogenous electric fields that guide cellular behaviours. Power management circuits are therefore required to condition the harvested energy into a therapeutically relevant waveform. The mismatch between TENG output characteristics and the electrical parameters that drive cellular responses illustrates a broader engineering challenge. While the raw output of triboelectric and piezoelectric harvesters is typically high-voltage (tens to hundreds of volts) and pulsed, the cellular responses discussed in the following section are predominantly elicited by low-voltage DC or low-frequency AC fields in the range of 40–200 mV/mm and 0.5–100 Hz (49). Effective translation of self-powered materials into clinical wound care therefore requires not only material innovation but also careful circuit design to condition the output into a biologically relevant waveform. This parameter-matching problem remains a critical challenge that has received limited attention in the development of self-powered wound dressings. The amplitude and the frequency of these outputs are difficult to regulate in a precise way. The main scientific and technical problem of this domain is to find ways, by material engineering or by circuit architecture, to properly manage and transform this irregular mechanical energy in a way to produce a stable electrical signal for the bioelectrical stimulation needs.

### 3.3 Integrated multifunctional electroactive materials: synergistic integration of electroactive functionality with other functional components

A growing amount of work is trying to combine therapies on the basis of electric fields with drug treatments, with immune system regulation and with control of the wound fluid all at once. In this way, all of the different related pathological problems that are found in chronic wounds, like an ongoing infection, overproduction of exudate, prolonged inflammatory response, lack of blood flow and oxygen and lack of important growth factors, are treated all at once.

As for the idea of dual purpose synergy, a study by Li and co-workers in 2023 combined the clinically effective negative pressure wound therapy (NPWT) with the triboelectric nanogenerator (TENG) system. In the configuration, the rhythmical mechanical deformations generated by the NPWT process were directly taken by the TENG part and converted to electrical power. Thus a constant and safe additional electric field was built up over the wound site. Such a strategy solved the possible concern that the traditional NPWT may weaken the intrinsic wound electric field of the body, while at the same time achieved several therapeutic purposes, such as physical drainage, the alleviation of tissue swelling, and the application of a healing-promoting electric field, all in one non-invasive therapeutic strategy (57). In the part of multi-functional combination, Liu and co-workers developed a self-repairing biomimetic neuro-immuno-regulatory (BNIR) platform by the smart assembly of MXene nanosheets, decellularized adipose tissue (DAT) and basic fibroblast growth factor (bFGF). This platform integrates the control of exudate, electrical conductivity, antioxidant properties, regulated growth factor delivery and immunomodulatory function in the same structure. In a diabetic wound model, the BNIR system promoted a phenotypic switch of macrophages from M1 to M2, which significantly accelerated the resolution of inflammation, the formation of new blood vessels and the organised deposition of collagen (58). A recent study developed a nanofibrous scaffold with an asymmetric design composed of hydrophilic and hydrophobic layers. By creating an asymmetric wetting gradient, this scaffold allowed the movement of the wound exudate in a directional way. At the same time, it activated the Cu/Zn galvanic pairs to create a continuous microcurrent of about 465 mV, which triggered the release of curcumin. This is a way to have at the same time active exudate handling, stimulus-responsive drug delivery and intrinsic electrical activation in a synergistic manner (59).

The examples presented here show how the integrated approaches have evolved from the crudest physical mixture to more advanced functional synergy. However, most of the composite materials discussed so far work in open-loop with a fixed capability: once manufactured, the electrical stimulation settings and the drug delivery modes are fixed and cannot be changed with real time, varying conditions of the healing process. A future direction is the development of closed loop intelligent wound covering. In 2025, a work appearing on Nature Communications presents a self powered, fully breathable, skin conforming stretchable electronic bandage called iSAFE. This device provides continuous monitoring of the status of the wound with a responsive local administration of the medication. The system was checked with clinical trials with different types of wound in patients from 18 to 95 (60). In the same year, another team presents a compact wireless system, a-Heal, to monitor wound healing. The images are taken of the wound by a wearable apparatus, and the stage of the recovery is evaluated with some machine learning techniques. After the stage of recovery is assessed, the system gives either electrical stimulation or a medicine, which forms a completely closed-loop control (61). Zhu and researchers made a Janus style multifunctional dressing which can do infection surveillance with quick pH response. It gives the potent electric field produced by ultrasound activated piezoelectric particles to kill bacteria, and after that it helps the tissue to be regenerated with low intensity self powered electric field produced by the motion of body (62).

These advanced material designs with high integration have also improved the overall therapeutic outcomes for wound healing; however, their complexity also brings new challenges. The main difficulty is the big increase of the complexity for both the material system and the manufacturing process. First of all, the organized combination of different active ingredients, and the guarantee of their stable incorporation in the material, and the guarantee of their behaving in a responsive way as needed, put strong requirements on the synthesis and the production methods. An additional worry is that the presence of multiple functional elements at the same time could lead to unexpected interactions. For instance, the sustained release of the metallic ions could harm the efficiency of the movement of charge in the conductive framework, and the shifts of the local pH that happen during the electrical stimulation could modify the rate at which the drugs are released (59, 62). Therefore, to achieve the integration is not only to add functions together. Instead, it should be based on a full understanding of the complex interaction processes that take place at the interface between the materials and the biological systems. At the level of structural design, approaches such as spatial compartmentalization, temporal separation, and selective activation in response to specific stimuli have to be taken into account. As materials science, bioelectronics and artificial intelligence continue to merge, the way to go for the next generation of intelligent wound dressings is to create closed-loop systems that are able to detect the wound conditions and flexibly modify the treatment approaches.

To facilitate comparison, the key characteristics of the three material categories are summarized in Table 1.

**Table 1:** Comparison of the three categories of electroactive materials for wound healing.

Feature	Conductive Hydrogels	Self-Powered Materials	Integrated Multifunctional Systems
Primary mechanism	Passive transmission of endogenous or applied electric fields	Active generation of electric fields from ambient energy	Combined electrical stimulation with auxiliary therapeutic functions
External power requirement	Required for active stimulation; not required for passive conduction	Not required	Depends on design
Key advantage	Soft, tissue-conforming interface; low impedance contact	Eliminates external power supply; wearable	Addresses multiple pathological factors simultaneously
Key limitation	Conductivity decay with hydrogel swelling/degradation; power dependency for active mode	Pulsatile, irregular output; power management required	High complexity in fabrication and quality control; potential component interference
Representative materials	PANI, PPy, PEDOT:PSS, MXene, CNT, graphene–hydrogel composites	PVDF-based piezoelectric films, TENG patches	Electroactive composite scaffolds with drug delivery, exudate management, or immunomodulatory functions
Development stage	Preclinical (animal models)	Preclinical (animal models)	Preclinical (animal models); some systems reaching large-animal or early clinical testing
Key references	(38, 45, 46)	(49, 52, 53)	(57, 58, 60)

The relationship between endogenous electric fields and electroactive material strategies is schematically illustrated in Figure 1.

**Figure 1:**
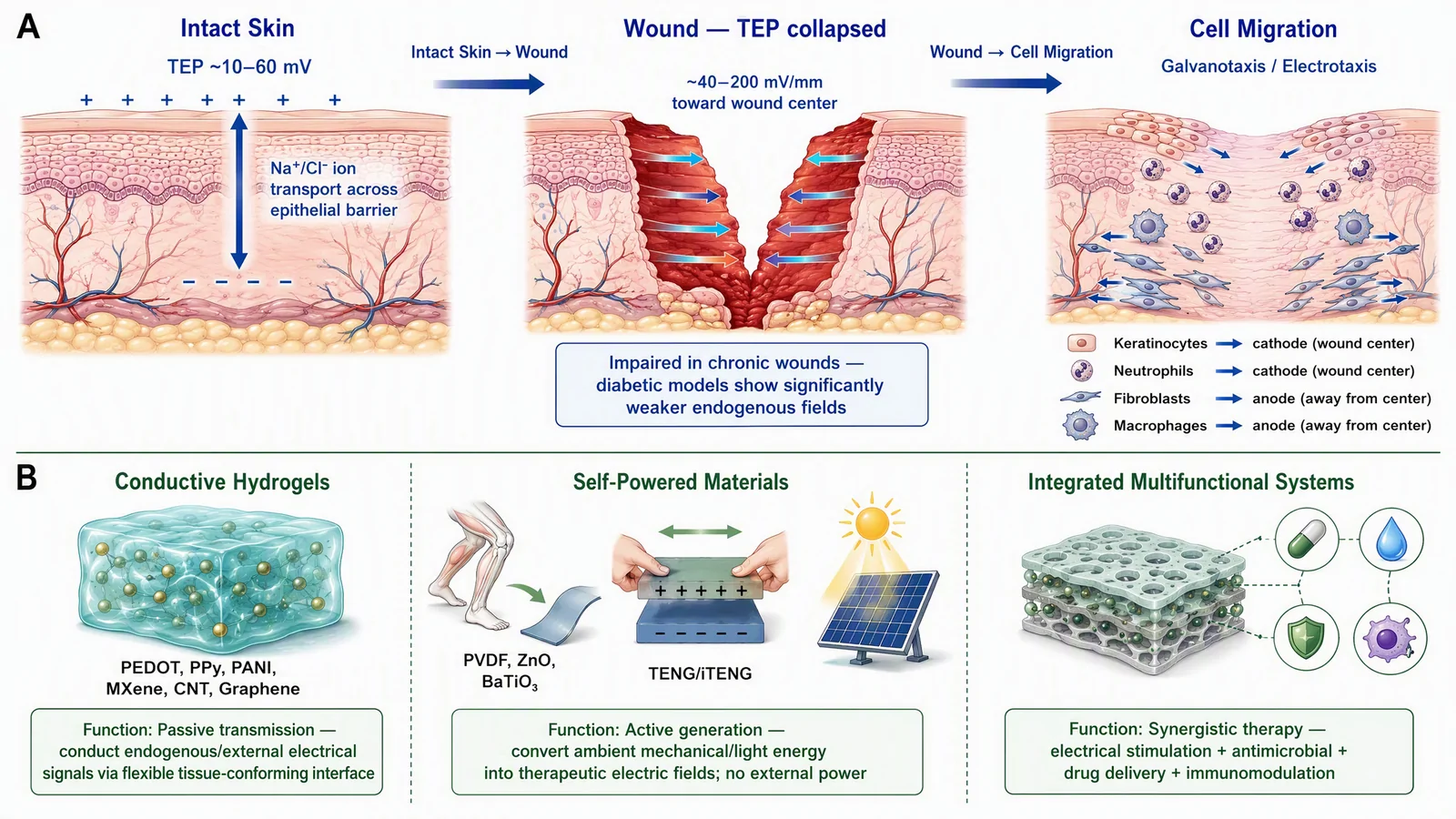
Overview of endogenous electric fields and electroactive material strategies for wound healing. **(A)** Endogenous electric field. Intact skin maintains a transepithelial potential (TEP) of ∼10–60 mV. Upon injury, TEP collapses, generating a lateral DC field (∼40–200 mV/mm) that guides cell migration toward the wound center. This field is impaired in chronic wounds. **(B)** Electroactive material classification. The electroactive materials discussed in this review are organized into three classes according to their primary mode of electrical action: conductive hydrogels (passive transmission), self-powered materials (active generation), and integrated multifunctional systems (combined functions).

## 4 Mechanisms by which electric fields promote wound healing

The three types of material strategies described above are different in their use in practice, but they all rely on the fact that electric fields can have an effect on the process of wound repair. Hence, it is important to understand what the exact molecular, cellular and tissue level mechanisms are by which electric fields speed up healing, in order to improve the material engineering and to find the right parameters of electrical stimulation. In the following we describe these mechanisms following the chronology of the stages of wound healing. We cover in five points the following: creation of directional cues, modulation of the inflammatory stage, control of the proliferative stage, control of the remodeling stage and antimicrobial activity.

### 4.1 Establishment of directional signals

The influence of electric fields on the healing process of wounds starts at the cellular level with the detection of electrical orientation. By means of gene knockout methodologies, the work of Zhao et al. showed that the electrotactic responses of the cells are co-regulated by PI3K*γ* and PTEN. When the cells are put in a direct-current electric field, PI3K*γ* localizes to the region of the plasma membrane oriented to the cathode, while PTEN is on the main anodal side. This asymmetric localization allows for the selective assembly of actin filaments on the cathodal side, which gives the molecular push for the persistent migration towards the cathode. The directional migratory cue induced by the electric field is completely removed when PI3K*γ* is genetically removed, while the electrotactic behaviour is strongly amplified after PTEN deletion (34). This regulatory pathway is functional not only in the neutrophils and macrophages present in the inflammatory stage of the repair, but also in the keratinocytes (33), in the stem cells of the epidermis (63) and in the fibroblasts (64) during the growth stage.

There is also a variety of other signaling molecules and ion channels that are additionally implicated in the detection of electric fields by the cells, and in the ensuing transmission of signals. In the sensing of electric fields, the calcium ions play the role of secondary messengers: an exposure to the electrical stimuli can therefore lead to a short increase of the calcium level inside the cells, that will afterwards turn on proteins that use calcium and are involved in the restructuring of the cytoskeleton (33). The transduction of the electrical signal may also involve other signaling molecules, such as the epidermal growth factor receptor (EGFR) and the integrin-Rac1 axis which will in the end feed all to the PI3 K/Akt and the mitogen-activated protein kinase/extracellular signal-regulated kinase (MAPK/ERK) cascades down (65, 66). There are also some interactions between the pathways for the electric field signaling and those for the sensing of the mechanical stimuli. Kim and colleagues demonstrated that the micro-electric field generated by a self-powered patch could activate the mechanosensitive ion channel Piezo1, leading to calcium influx that promotes cell migration and angiogenesis (67).

It should be noted that the mechanistic framework summarized above—particularly the PI3K*γ*/PTEN polarity pathway and Piezo1-mediated calcium signaling—has been primarily established through studies using acute wound models in healthy rodents or *in vitro* cell cultures. Chronic human wounds, such as diabetic ulcers, are characterized by persistent hypoxia, ischemia, and a dysregulated inflammatory milieu that may alter cellular responsiveness to electric fields. Notably, diabetic wounds generate significantly weaker endogenous electric signals than healthy tissues (35), raising the question of whether the molecular machinery for electrotaxis remains fully functional under pathological conditions. Systematic studies using chronic wound-relevant models are needed to validate these pathways in the chronic wound microenvironment.

From a quantitative perspective, the threshold for electrotactic response varies across cell types. Keratinocytes require field strengths above approximately 50 mV/mm for directional migration, with migration rates increasing with field strength (31, 33). Fibroblasts, by contrast, are responsive to field strengths in the range of 50–100 mV/mm (64), suggesting that different cell types have evolved distinct sensitivity thresholds to electric fields. These differences may reflect the specific roles of each cell type in the wound healing cascade—keratinocytes must traverse the wound bed during re-epithelialization, while fibroblasts are responsible for matrix remodeling and contraction. Macrophages respond to fields as low as 5 mV/mm (68), while endothelial cell responses vary by vascular origin: HUVECs respond to fields as low as 50 mV/mm, whereas HMECs require 100 mV/mm for directed migration (69). These differences suggest that different cell types have evolved distinct sensitivity thresholds to electric fields. Understanding these threshold differences is essential for designing stimulation protocols that selectively target specific cell populations without causing off-target effects.

### 4.2 Regulation of the inflammatory phase

In the inflammatory stage electric fields affect the biological processes by different ways: for example, guiding movement of the immune cells, the transition in the macrophages phenotypes, the release of inflammatory factors. In the literature it is shown, that for example, under direct-current electric field, which is similar to the physiological one, the neutrophils and human peripheral blood lymphocytes have a directed movement towards the wound site, which is the cathode (33, 70). The electrotactic response of the macrophage depends on the maturation of the cell: the differentiated macrophage moves to the anode and the capability of these cells to engulf the particles is highly increased by the action of the electric field, and this increase is related to the activation of the PI3 K and ERK pathways and the mobilisation of the calcium inside the cell. On the contrary, the precursors of the monocytes migrate in the opposite direction moving towards the cathode. The threshold for this response is low: differentiated macrophages are directed anodally by fields as low as 5 mV/mm, with effects peaking at approximately 300 mV/mm (68). This threshold is substantially lower than that of keratinocytes (>50 mV/mm) and fibroblasts (50–100 mV/mm), indicating that macrophages are among the most electrically responsive cell types in the wound microenvironment.

With respect to the resolution of inflammation, the electrical fields can lead to the shift of the macrophages from a pro-inflammatory M1 state to a pro-healing M2 state. In a work involving a murine wound model, it was found that after three days of exposition to the electric field the local M1 to M2 ratio was decreased by 30.6%,with a trend toward a 24.2% increase in re-epithelialization compared with the control group. This change occurred at the same time of lower levels of the pro-inflammatory cytokines TNF-α and IL-1β and higher levels of the anti-inflammatory cytokine IL-10 (71). Other *in vitro* studies showed that with the application of the direct-current stimulation of 100 mV/mm for one hour daily during three days, the expression of the genes of the M2 markers, namely IL10, CD163 and PPARG, in M0 macrophages was increased. For M1 macrophages, with the identical stimulation regimen the expression of the M2 markers IL10, TGM2 and CD206 was increased, while the expression of the M1 marker CD86 and the release of IL-1β and IL-6 was decreased (72). Finally, the electric fields can also directly act on the expression and the secretion of inflammatory mediators. Lu et al. found that after HaCaT cells were kept in a direct-current electric field of 200 mV/mm, the Erk1/2 signaling cascade was activated, and the production of the pro-healing cytokines, e.g., granulocyte-macrophage colony-stimulating factor (GM-CSF), was markedly increased (73). The time course of stimulation also differs between these two regimens. Continuous 3-day exposure reduced the M1/M2 ratio by 30.6%, whereas 1-hour daily pulses over three days also achieved M2 polarization (71, 72). Both regimens achieved M2-like phenotypes, but the different temporal patterns indicate that cumulative exposure, rather than the distinction between continuous and intermittent stimulation itself, may be the key determinant of macrophage phenotype switching. This observation has practical implications for device design, as intermittent stimulation may be more compatible with wearable or self-powered systems that generate pulsatile outputs, whereas continuous stimulation may require more stable power sources.

### 4.3 Regulation of the proliferative phase

During the proliferative stage, keratinocytes move to facilitate the re-epithelialization, fibroblasts multiply and specialize in order to colonize the wound matrix, and endothelial cells construct fresh vascular networks. Concerning the re-epithelialization, both keratinocytes and epidermal stem cells show the movement toward the cathode when they are submitted to physiological direct-current electric fields (33, 63). It is observed that the migration of keratinocytes is not a random and isolated cellular activity; on the contrary, the keratinocytes migrate as extensive cellular sheets collectively toward the negative electrode. This form of electrotaxis is a migration with greater migration efficiency and better directionality with respect to the migration of single cells (74). Moreover, the use of direct-current electrical stimulation promotes the proliferation of human HaCaT keratinocytes, upregulates the expression levels of keratins K5 and K14, and mediates the pro-keratinization effects through the phosphorylation of the ERK1/2 and p38 mitogen-activated protein kinase (p38 MAPK) signaling pathways (75).

For the development of the granulation tissue, the migration of the fibroblasts is towards the anode by the presence of the electric field. Application of electrical stimulation increases the production of fibroblast growth factor-1 (FGF-1) and fibroblast growth factor-2 (FGF-2) by these cells. It also increases the levels of the alpha-smooth muscle actin (*α*-SMA) that help them transform into myofibroblasts (76). A detailed analysis has been presented by Wang et al. for the signaling pathways that are responsible for this differentiation induced by the pulsed electrical stimulation. They showed that in the fibroblasts that have been subjected to electrical stimulation, the levels of transforming growth factor-beta1 (TGF-*β*1) and phosphorylated ERK are also increased. Also, nuclear factor-kappaB (NF-*κ*B) is accumulated inside the nucleus and the transcription of the *α*-SMA gene is activated. The same investigation has also shown that these activated fibroblasts kept the *α*-SMA expression elevated over several cell divisions, and a large number of human myofibroblasts could be identified also 15 days after the subcutaneous implantation (77). Furthermore, Yang et al. found that the application of electric fields induces the transformation of fibroblasts into myofibroblasts, which is related to a large amount of collagen matrix deposition. This newly formed collagen and its network structure have been found to enhance the epidermal cell migration along the electrical direction, thus effectively promoting the re-epithelialization efficiency (78).

In terms of angiogenesis, the endothelial cells show also polarized reactions to the electrical field that is different according to the vascular source of the endothelial cells: the endothelial cells from the microvessels move towards the negative electrode, while the endothelial cells from the larger vessels move towards the positive electrode (79). Direct-current electric fields with a physiological relevance can directly cause oriented migration and the development of tubular structures in the endothelial cells by the engagement of the vascular endothelial growth factor (VEGF) receptor signaling cascade, even at the intensity of 75 to 100 mV/mm (80). Electric fields can also stimulate the capillary morphogenesis in the microvascular endothelial cells through the MAPK/ERK pathway that is frequency sensitive and does not require VEGF to bind to VEGFR2 (81). At the same time, low intensity electric fields can directly stimulate the Piezo1 channels, and in consequence there is an influx of calcium ions that encourages the angiogenic processes (67). On the paracrine level, the electrical stimulation has the ability to activate also the MAPK/ERK signaling cascade and to enhance the secretion of fibroblast growth factor-2 (FGF2) by the fibroblasts, thus enhancing the movement of the endothelial cells and their ability to form new blood vessels through a paracrine mechanism (82). Also, direct-current electric fields that simulate natural physiological conditions can increase the expression of the chemokine receptors CXCR4 and CXCR2 on the endothelial cells, and upregulate the phosphorylation levels of the associated chemokines, which improves the capacity of the endothelial cells to detect and respond to the chemotactic cues (69). In the same study, HUVECs responded to fields as low as 50 mV/mm, while HMECs required 100 mV/mm for directed migration. Mitosis was stimulated at 50 mV/mm for HMECs and at 150 mV/mm for HUVECs, with the cleavage plane becoming oriented perpendicular to the field at 200 mV/mm for both cell types (69). These data indicate that microvascular endothelial cells, which are the primary source of new vessels during wound repair, require a stronger signal to initiate migration than macrovascular cells, and that field strengths above 200 mV/mm may impose spatial constraints on cell division.

### 4.4 Regulation of the remodeling phase

In the remodeling stage the extracellular matrix (ECM) is being reorganized and the scar is maturing. In the newly developed granulation tissue, the initially delicate type III collagen fibrils are replaced by more robust type I collagen fibers. However, if there is an overabundance of collagen accumulation and irregular fiber arrangement, this could lead to hypertrophic scars or keloids (83). In contrast to the proliferative phase, which stimulates the collagen production, the main function of the electric field in this period is to prevent an excessive collagen accumulation and to stimulate the systematic reorganization of the ECM.

Gouarderes and colleagues investigated the effect of pulsed electric fields (PEFs) on the remodeling of the extracellular matrix (ECM) in the tissue-engineered human dermal substitute. They showed that PEFs for duration in the microsecond and millisecond range have combined effect on the four different levels. Shortly after the electrical stimulation, in a four hour window, the expression of genes for production of the procollagens, and two enzymes that are important for the cross linking and maturation of the ECM, transglutaminase 2 and lysyl oxidase-like protein, is downregulated. In the next week there is a temporary reduction in both the synthesis of the procollagen and the total hydroxyproline contained in the tissue. The activity of the matrix metalloproteinases (MMPs) is still high for at least 48 h, the process is driven by the generation of reactive oxygen species (ROS). At the same time, the expression of transforming growth factor-*β*1 (TGF-*β*1) is also down-regulated (84). As for the signaling mechanisms, in the study of Wang and coworkers, after pulsed electric field stimulation at the intensity of 50 and 100 mV/mm, there was a significant increase in the level of phosphorylated Smad2 and Smad3 in fibroblasts. And this increase is associated with the enhanced secretion of fibroblast growth factor-2 (FGF-2) and the increased expression of *α*-SMA. These results indicate that Smad signaling pathway may be involved in how the electric field regulates the activity of fibroblasts (85).

The above described regulatory mechanisms at the transcriptional and enzymatic level have been experimentally verified, both *in vitro* and *in vivo*. For example, in Kim and colleagues, the continuous direct-current electric field was obtained with a wearable, multilayer electret patch. *In vitro* experiments yield a suppression of the expression of type I collagen by 90% and a reduction in the level of *α*-SMA by 44%. The corresponding *in vivo* experiments show a reduction in the populations of myofibroblasts by 20% with respect to the control (86). Recently, it was found that low-frequency alternating current electrical stimulation downregulates the synthesis of type I procollagen and upregulates the expression of matrix metalloproteinase-1 (MMP-1) in human dermal fibroblasts. The effect is probably associated with changes in the expression level of the mechanosensitive ion channel PIEZO2 and the membrane microdomain associated protein CAVIN2 (87).

### 4.5 Antibacterial effects

Apart from the afore-mentioned regulation effects of the host repair cells, electric fields also directly inhibit the pathogenic microorganisms infecting the wound. The delayed healing of chronic wounds is strongly related to the formation of bacterial biofilms. A systematic review and meta-analysis have reported a high detection rate of biofilms in chronic wounds (88).

Ashrafi and co-workers performed a direct experimental verification of the biofilm-inhibiting effects of electrical stimulation using an *ex vivo* human skin wound model. For biofilms of Staphylococcus aureus, a decrease was observed both in the number of viable bacteria and in the total biofilm mass when the biofilm was subjected to electrical stimulation. In the case of Pseudomonas aeruginosa biofilms, the treatment produced in the same way a reduction in bacterial load, metabolic activity, and total biomass, with a statistically significant reduction of greater than 60% in bacterial viability (*p* < 0.05). This work also showed that the composition of the volatile organic compounds emitted by the biofilms subjected to electrical stimulation showed characteristic changes, which can be considered to function as a non-invasive indicator for tracking the therapeutic outcome (89). Beyond *in vitro* and animal studies, preclinical evidence from a porcine model of polymicrobial biofilm infection demonstrated that a wireless electroceutical dressing generating a weak electric field upon contact with wound fluid prevented biofilm formation and disrupted established Pseudomonas aeruginosa–Acinetobacter baumannii biofilms over 56 days of treatment. The dressing also blunted bacterial quorum sensing gene expression and suppressed NF-*κ*B-driven inflammatory responses (90). Mechanistically, the antibacterial effect of this platform has been attributed to the Ag/Zn redox couple-generated electric field and associated reactive oxygen species, whereas the silver component alone exhibited no significant anti-biofilm activity (91).

### 4.6 Summary

Collectively, the contribution of electric fields to wound healing is a multi-tiered biophysical process that includes the whole process of tissue repair, starting from the inflammation and going through the proliferation to the remodeling stages. From the detection of the signals to the reconstruction of the tissue, the electric fields provide orientational guidance to the repair cells by, among other mechanisms, the PI3K*γ*/PTEN polarity signaling route and the Piezo1 ion channel. In the inflammatory stage, they guide the recruitment of immune cells and promote the shift in the macrophage phenotype from M1 to M2, which accelerates the resolution of inflammation. In the proliferative stage, they promote the differentiation of myofibroblasts by the TGF-*β*1/ERK/NF-*κ*B signaling axis and support the formation of new blood vessels through several synergistic pathways. In the remodeling phase, the electric fields stimulate the matrix metalloproteinases (MMPs) at the same time that they reduce the expression of TGF-*β*1, which limits the excessive accumulation of collagens and refines the reorganization of the extracellular matrix (ECM). Moreover, the electric fields demonstrate antimicrobial effects by disrupting bacterial biofilms. This regulatory mechanism, which spans the entire recovery continuum and affects as many cell types involved in the repair, as well as their signaling cascades, is a capability that single growth factors or specific pharmacological treatments are not able to replicate.

To further illustrate the diversity of electrical stimulation parameters and their corresponding biological effects, key representative studies are summarized in Table 2.

**Table 2:** Representative electrical stimulation parameters and biological effects in wound healing studies*.*

Stimulation type	Parameters	Cell/tissue type	Key biological effect	References
DC EF	100–200 mV/mm	Mouse fibroblasts, keratinocytes	PI3K*γ*/PTEN-mediated electrotaxis; directional migration	(34)
DC EF	40–200 mV/mm	Neutrophils, macrophages, keratinocytes, fibroblasts	Cell-type-specific directed migration (cathode vs. anode)	(33)
Pulsed EF	50–100 mV/mm	Human dermal fibroblasts	TGF-*β*1/ERK/NF-*κ*B activation; myofibroblast differentiation; *α*-SMA ↑	(77)
DC EF	100–300 mV/mm	Human dermal fibroblasts	FGF-1/FGF-2 secretion ↑; α-SMA ↑; myofibroblast transdifferentiation	(76)
Self-powered micro-EF	Self-generated via motion	Endothelial cells	Piezo1 activation; Ca^2^⁺ influx; angiogenesis ↑	(67)
DC EF (wearable)	3 days continuous	Murine wound model	M1/M2 ratio ↓ 30.6%; re-epithelization ↑ 24.2% (trend); TNF-α/IL-1β ↓	(71)
DC EF	100 mV/mm, 1 h/day × 3 days	THP-1 macrophages (M0, M1, M2)	M2 markers (IL10, CD163, PPARG) ↑; CD86 ↓; IL-1β/IL-6 ↓ (*p* < 0.05)	(72)
ES	—	Fibroblasts, HUVECs	FGF2 secretion ↑; MAPK/ERK pathway activation; angiogenesis ↑	(82)
DC EF	75–100 mV/mm	Microvascular/macrovascular ECs	VEGF receptor signaling; oriented migration (cathode vs. anode)	(79)
ES	—	S. aureus, P. aeruginosa biofilms	Viable bacteria ↓ > 60%; biofilm mass ↓; metabolic activity ↓ (all *p* < 0.05)	(89)
WED (Ag/Zn)	0.3–0.9 V	P. aeruginosa biofilm	Ag/Zn redox couple EF; biofilm disruption; ROS generation	(91)

DC, direct current; EF, electric field; ES, electrical stimulation; WED, wireless electroceutical dressing; HUVECs, human umbilical vein endothelial cells; ECs, endothelial cells; FGF, fibroblast growth factor; VEGF, vascular endothelial growth factor; TGF-β, transforming growth factor-β; NF-κB, nuclear factor kappa B; α-SMA, alpha-smooth muscle actin; ROS, reactive oxygen species.

Fibroblasts respond to a broad range of DC fields (25–300 mV/mm), with migration toward the anode and differentiation into myofibroblasts via TGF-*β*1/ERK/NF-*κ*B signaling. Pulsed stimulation at 50–100 mV/mm appears particularly effective for promoting myofibroblast differentiation, whereas continuous DC at 100–300 mV/mm enhances FGF-1/FGF-2 secretion (76, 77). The differentiation of fibroblasts into myofibroblasts is critical for wound contraction and matrix remodeling, yet the optimal stimulation regimen for this process remains to be systematically defined.

Keratinocytes exhibit cathodal migration in DC fields comparable to those measured *in vivo*, with migration rates increasing at field strengths above 50 mV/mm (31, 33). Importantly, higher field strengths may suppress proliferation, suggesting a relatively narrow therapeutic window for keratinocyte-targeted stimulation. This sensitivity underscores the need for precise parameter control in electroactive dressings designed to promote re-epithelialization.

Endothelial cells respond to DC fields as low as 75–100 mV/mm through VEGF receptor signaling and MAPK/ERK pathway activation, with Piezo1-mediated Ca^2^⁺ influx further promoting angiogenesis (67, 79, 80). The distinct migratory responses of microvascular versus macrovascular endothelial cells—cathodal versus anodal migration, respectively—further illustrate the cell-type specificity of electrotactic responses (79).

Immune cells show distinct parameter dependencies as well. Macrophage polarization toward the M2 phenotype was achieved with 100 mV/mm DC applied for 1 h daily over three days (72), while continuous 3-day exposure at similar field strengths reduced the M1/M2 ratio by 30.6% *in vivo* (71). These contrasting temporal regimens suggest that cumulative exposure—rather than continuous versus intermittent stimulation *per se*—may be the key determinant of macrophage phenotype switching, a finding with practical implications for device design.

Antimicrobial effects have been observed across a remarkably wide parameter range (from microampere-level currents to milliampere-level currents), suggesting that bacterial biofilms are broadly susceptible to electrical fields. However, the optimal parameters for biofilm disruption—particularly in the context of polymicrobial wound infections—remain to be established (89, 91).

Collectively, these observations indicate that there is no single optimal parameter set for electrical stimulation in wound healing. Rather, stimulation parameters should be tailored to the target cell type, the specific phase of healing, and the therapeutic goal. This cell-type-specific parameter landscape provides a quantitative framework for designing future studies and highlights the critical need for systematic parameter optimization—particularly when translating *in vitro* findings to *in vivo* wound healing applications. The heterogeneity of reported parameters across studies, as summarized in Table 2, also underscores the difficulty of establishing universal stimulation protocols and supports the ongoing efforts to develop closed-loop systems capable of adapting stimulation in real time based on wound stage and cellular responses.

The molecular and cellular mechanisms by which electric fields regulate each phase of wound healing are schematically illustrated in Figure 2.

**Figure 2:**
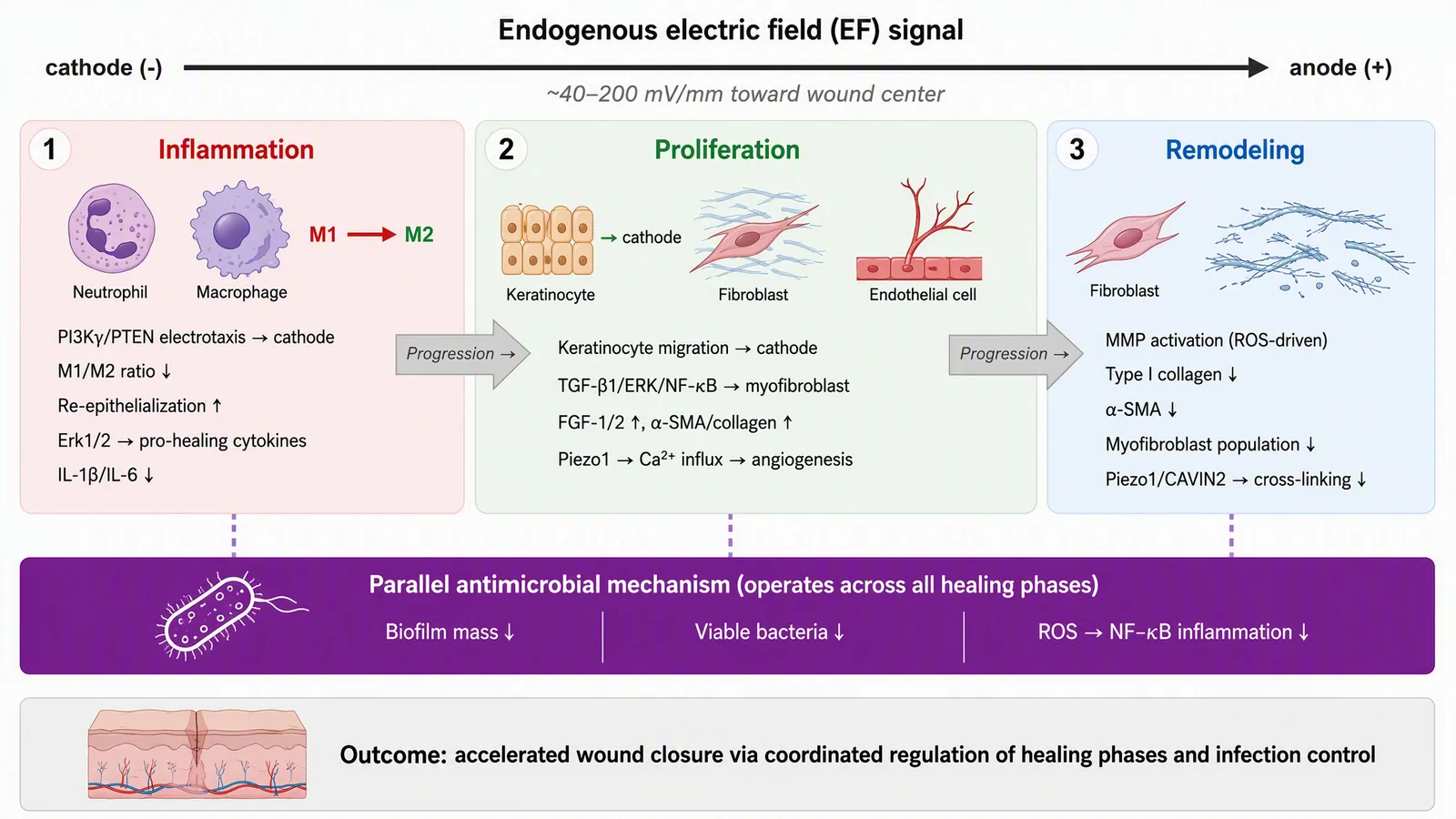
Molecular and cellular mechanisms of electric field-mediated wound healing. The schematic summarizes signaling pathways and cellular responses during inflammation, proliferation, remodeling, and antimicrobial phases. Arrows indicate upregulation (↑) or downregulation (↓). PI3K*γ*, phosphatidylinositol-3-OH kinase-*γ*; PTEN, phosphatase and tensin homolog; TGF-*β*1, transforming growth factor-*β*1; ERK, extracellular signal-regulated kinase; NF-*κ*B, nuclear factor *κ*B; *α*-SMA, *α*-smooth muscle actin; MMP, matrix metalloproteinase; VEGF, vascular endothelial growth factor; FGF, fibroblast growth factor; ROS, reactive oxygen species.

## 5 Conclusions and perspectives

The deep integration of materials science with bioelectronics is driving a radical change in the way that therapeutic approaches using electric fields for wound management are shifting from passive intervention to active biological control. In this work we have methodically reviewed the advances in conductive hydrogel systems, autonomously powered materials, and combined multifunctional platforms. We have shown a core logic of development: the crucial importance of new electroactive materials is that they can act to dynamically simulate, boost and intelligently tune the endogenous bioelectric microenvironment in wounds. It allows coordinated, multi-stage interventions timed to the different phases of tissue repair. In this respect it provides a unique biophysical regulatory perspective that represents a promising strategy for overcoming the limitations of conventional drug-based or growth factor therapies, namely their focus on single targets and inability to reproduce the complex physiological healing sequences.

The creation of biomimetic interfaces allows the conductive hydrogels to effectively and biocompatibly transmit electrical signals. The self-powered materials are clever in the way they convert ambient energy into therapeutic electric fields: this is a further step towards therapies that do not need external power. On the other hand, the integrated multifunctional materials are just beginning to show the great promise of synergistic treatments based on electrical stimulation. All these approaches share the common aim of restoring or improving the disrupted bioelectric signaling network typical of chronic wounds from different complementary dimensions.

Although these prospects are promising, this technology still faces several challenges on the way to routine clinical application. First, the contradiction between standardized and individualized electrical stimulation parameters must be resolved. The parameter heterogeneity documented in Table 2—ranging from 40 mV/mm to 300 mV/mm in field strength, from minutes to days in duration, and from DC to pulsed to AC waveforms—underscores the difficulty of establishing a universal stimulation protocol. Moreover, the cell-type-specific parameter preferences discussed in Section 4 indicate that a single “optimal” parameter set is unlikely to be applicable across all cell types and healing stages. Dynamic parameter databases adapted to the wound type, the healing stage, and the target cell population need to be established to guide both material design and clinical application. Second, the long-term durability and biological safety of the materials, such as their stability in the moist wound environment and the decay of electrical conductivity over time, require more systematic evaluation. Third, most of the current systems operate in a pre-defined, non-responsive open-loop mode, and at present they are unable to alter their action in real time in response to the changing physiological conditions in the wound site. Fourth, manufacturing scalability, sterilization compatibility, shelf-life stability, and regulatory approval pathways remain largely unexplored for most electroactive wound materials; while some preclinical studies have been conducted in large-animal models (90), systematic evaluation across different wound types and direct comparative studies with standard care remain limited. Bridging these translational gaps will require close collaboration between materials scientists, wound care clinicians, and regulatory bodies.

The next step will be to build closed-loop intelligent healing platforms with sensing, decision-making, and responsive functionalities. This would represent a significant shift in the design paradigm. Recent proof-of-concept studies have demonstrated the feasibility of integrating flexible sensors with wireless stimulation circuits (60, 61), and machine learning-assisted wound assessment has shown promise for automated stage classification. However, translating these approaches into clinically viable systems remains a significant challenge. The initial generation of practical closed-loop systems will likely focus on relatively simple feedback mechanisms—such as pH- or temperature-triggered stimulation adjustment—while more sophisticated machine learning-based decision-making belongs to a longer-term horizon. Future systems will go beyond simple electrical stimulation towards multi-modal approaches that combine programmable electric fields with adjustable mechanical forces (e.g., dynamic stress) and biochemical cues (e.g., spatiotemporally controlled delivery of cytokines) to temporally coordinate and precisely modulate specific cell types, such as fibroblasts, immune cells and vascular endothelial cells, going beyond simple on-demand drug release. At the same time, a much more comprehensive study of how electric fields control immune responses, e.g., macrophage polarization and lymphocyte recruitment, will help to build intelligent electroactive materials with built-in immunomodulatory capabilities, in order to fundamentally reverse the persistent inflammatory environment of chronic wounds.

To conclude, the general goal of wound healing approaches based on electric fields and new materials is to go beyond the simple replacement of a material and to aim at the real functional replacement. If we can combine in the same flexible electronic component, a multi-sensor system and some kind of artificial intelligence, it is possible to build bio-integrated platform that could interpret the biological signal of a wound and bring an intelligent, adaptive intervention. This could allow to go from the standardized wound covering to a covering that can be adapted on the fly: it could be an evolution which can represent a leap in the clinical care of the persistent wound.
